# Augmented reality advertising and college students' interest in the extreme sports: Moderating role of innovation resistance and health consciousness

**DOI:** 10.3389/fpubh.2022.978389

**Published:** 2022-08-24

**Authors:** Shaoqiong Zhang, Ningning He

**Affiliations:** ^1^School of Physical Education, Hubei University, Wuhan, China; ^2^Hubei Leisure Sports Development Research Center, Wuhan, China; ^3^Faculty for Physical Education, Shanghai International Studies University, Shanghai, China; ^4^School of Journalism and Communication, Shanghai International Studies University, Shanghai, China

**Keywords:** mental health, health consciousness, augmented reality advertising, extreme sport, innovation resistance, college student attitudes

## Abstract

Advertising and promotions are the most utilized types of augmented reality (AR) activations for marketers across all industries. The same is true for the sports industry. This form of augmented reality is meant to bring attention to the organization through a novel technology such as AR. Recently, a lack of interest among students in extreme sports has been attributed to a lack of professional advertising and marketing innovation. This situation requires the attention of researchers, and this study investigates the impact of augmented reality advertising on college students' interest in extreme sports, specifically in China. The article also investigates the moderating role of innovation resistance and health consciousness in the relationship between augmented reality advertising and college students' interest in extreme sports in China. Students actively participating in sports were selected using the purposive sampling technique, and AMOS was used for data analysis. According to the findings, augmented reality advertising positively correlates with interest in extreme sports. The findings also revealed that innovation resistance and health consciousness significantly moderated college students' interest in extreme sports and augmented reality advertising. This research assists regulators in developing regulations to increase interest in extreme sports through augmented reality advertising and innovation adoption.

## Introduction

Today, the improving lifestyle has increased passion and interest in sports, making the sports industry one of the country's leading national economic sustainable industries (1). Because of its rising fame, high revenue generation, and numerous career opportunities, the sports industry is highly valued and ranked globally. The sports industry is well-known in today's modern society for its numerous business opportunities (2). The social economy and the sports industry contribute to a sustainable economy's growth, and the sports industry operates under a socialist economic system (3).

The sports industry's strong influence is not limited to the sports world; it also influences the marketing campaigns of various products. Organizations spend a large portion of their profits on advertising their products or services to gain a competitive advantage through marketing. Aside from sports, the sports performer participates in various other activities, such as serving as an ambassador for various brands (4). Consumers today regard sports players as reputable experts rather than celebrities; thus, they are inspired by their choices (5).

Sports are played on international platforms, so sports people have an international following and are frequently approached in advertisements by international brands. Aside from marketing and branding for sports products (6), sports players are also advertised in various everyday products, creating a second-level impact on the young population, who are influenced by the personalities of these sports players (7). In China, Europe, Russia, and America, extreme sports are rated higher than traditional sports. Although many industries are essential to advertising worldwide, all extreme sports have a distinct position (8). Young people are the most common participants in extreme sports, as these sports emphasize entertainment, bravery, and trends (9). These extreme sports activities highlight the abilities of young players who eagerly want to demonstrate them and perform without limitations. Indoor extreme sports such as rock climbing, skateboarding, parkour, and roller skating are most popular in Chinese colleges and schools (10).

China has become a world factory. Firms from all around the globe approach China for their business. China not only manufactures the products but also ensures the deliverance of all the services that pertain to a product, like advertisement, supply chain, and product sale. The product sale is based on the advertisement policy of the firm. The firm all-round the globe hires sports individuals to make their advertisement succeed. China, one of the world's developed nations, is a bit lacking in this concern. One of the core reasons that China focuses more on manufacturing than an advertisement (2). China is not very good in one of the most-watched and liked sports around the globe, like football and cricket. This lack in the sports industry also affects the chines advertisement industry. One of the prime aims of this study was to investigate the usage of extreme sports for advertisement, particularly in China, where manufacturing is preferred over advertisement (3).

Extreme sports are activities in which the participant is subjected to enormous physical and mental challenges, such as speed, height, depth, and natural forces, in which dangers or extreme endurance are frequently involved. They are also known as action, lifestyle, and alternative sports. They are distinguished by a desire for physical prowess and a unique perspective on the world and oneself. Triathlon, BMX, base jumping, cliff jumping, skiing, and skydiving are a few examples (4). There are also bungee leaping and caving.

Sports sponsorship is the most visible example of how sports can be used in advertising and as a setting to convey marketing messages. On the contrary, sports are frequently used as a meta-linguistic tool to express specific meanings and appeals and achieve positioning. It is because advertising in sports contexts can influence how consumers perceive appeals, brands, and goods. In the 2000's, one of the most appealing topics was sports advertising, and everyone meant traditional sports because large corporations had little interest in extreme activities. Among many other things, the importance of assessing the success of advertising in traditional sports was emphasized during those years.

Nonetheless, this study highlights the importance of investigating the effectiveness of advertising in extreme sports. It was not necessary to specify that “sports” meant traditional sports when studying sports advertising in the 2000's (5). Because the terms “free sports,” “adventure sports,” “lifestyle sports,” “alternative sports,” and “action sports” were similar but sometimes contradictory, the phrase “extreme sport” was coined later.

**Figure 1:**
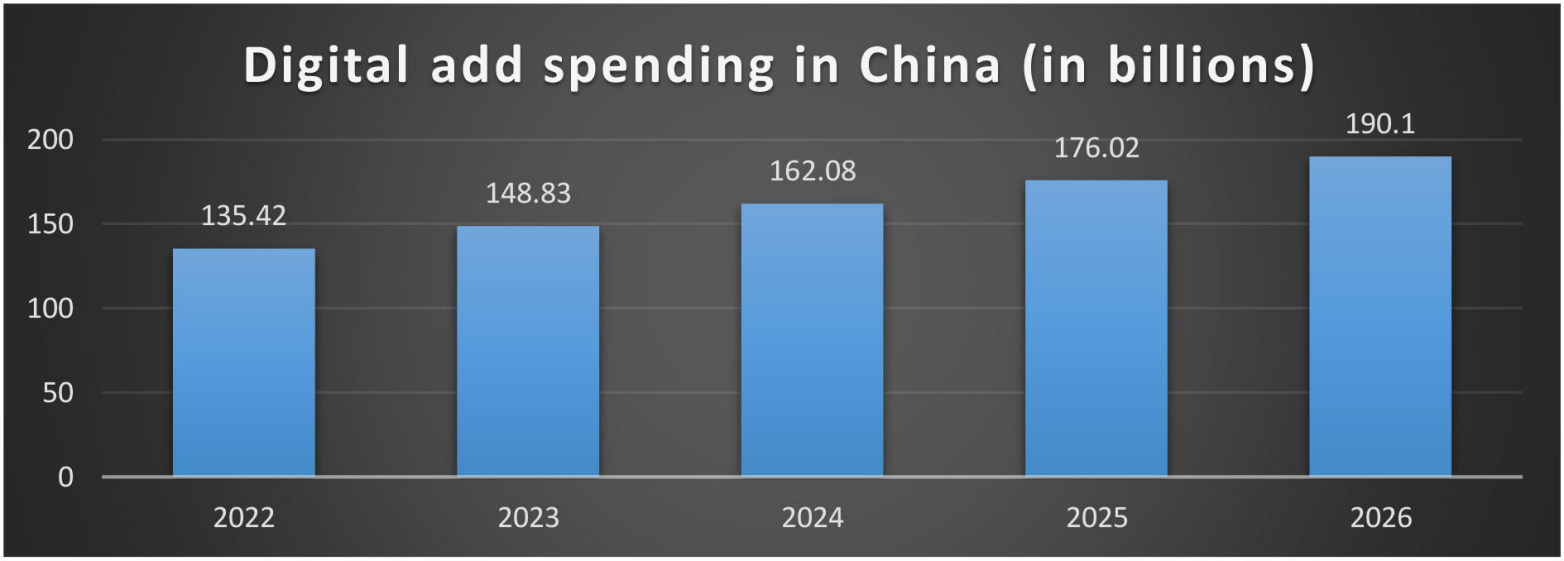
Digital advertisement spending in China.

However, during the 2000's, extreme sports experienced a massive increase in popularity. They have gone from a relatively new, mysterious, and niche phenomenon to a multi-billion dollar industry. For instance, the number of wakeboarders has increased by 32 % in the United States alone; there are over 6 million triathletes in the country, and BMX has become so popular that it is now an Olympic sport (6). Whether skydiving, base jumping, snowboarding, cliff diving, ice climbing, bungee jumping, caving, or triathlon, over 22 million individuals routinely engage in extreme sports in the United States alone each year (7). Annual extreme sports competitions like the X Games are attended by hundreds of thousands of people and are watched by 12.5% of adults and 8.6% of young adults in the United States, illustrating this global increase in popularity. The interest in extreme sports from globally recognized companies and media outlets, as well as the number of advertising campaigns placed in the setting of extreme sports, does not come as a surprise, and marketing and advertising spending in extreme sports have surged to catch up. For instance, Red Bull was a pioneer in promoting its goods in conjunction with extreme motorsports like snowboarding and snowboarding. However, many other companies, from Timex watches to Oakley eyewear, from Argon 18 bicycles to Vans clothing, promote their goods in conjunction with extreme sports (8). Marketers today increasingly use extreme sports as an advertising setting, drawing from their imagery to communicate values and visions and increase their customer base. Several brands also use extreme sports that frequently sell products unrelated to sports in their rush to find trendy new sports disciplines and attractive contexts. Lastly, those who like extreme sports typically have better purchasing power than ordinary Americans. It is reflected even in the advertising techniques of big manufacturers of standard sports equipment. The importance of advertising in China can be seen in the spending on digital advertising mentioned in Figure 1.

This study will fill some gaps in the literature, such as how the sports field is progressing with time as globalization exerts its impact; it has become one of the essential topics in recent history. As a result, it is one of the most critical topics studied, but several extreme sports-related factors must still be investigated to understand it better. Xinxin et al. (2–9) worked on the foundation of extreme sports tourism planning and development. In contrast, with a new data set, this study will work on the relationship between augmented reality advertising and interest in extreme sports from a Chinese perspective using the dual moderation effect, that is, health consciousness and innovation resistance. The model consists of augmented reality advertising, health consciousness, resistance to innovation, and interest in extreme sports, which have not previously been tested from the Chinese perspective with a new data set.

Moreover, Dikčius and Ilciukiene (4–10) conducted an overview of extreme sports comprehensively, whereas this study will check the association between augmented reality advertising and interest in extreme sports with dual moderation in China by selecting a new data set. Liu et al. (5–8) worked on the emotions of extreme sports, whereas this study will check extreme sports from the point of view of advertisement by employing the moderation effect of health consciousness and innovation resistance in China. Fathian et al. (6–11) checked the extreme sports ecological conceptualization.

In contrast, this study will check the effect of the advertisement on the interest in extreme sports with moderation effect in China by selecting a fresh data set. The significance of this study is that: (1) it will shine the spotlight on the one emerging sports category, that is, extreme sports, for the betterment of the sports industry in China; (2) it will help the Chinese extreme sports-related professionals to revamp their policies regarding the betterment of extreme sports to uplift the sports industry in China; and (3) it is a call for scholars to explore more aspects of extreme sports that cause improvise or down the extreme sports performance in China.

The structure of the study is divided into five chapters. The first chapter of the study will present the introduction. In the study's second phase, the evidence of augmented reality advertising, health consciousness, innovation resistance, and interest in extreme sports in light of past studies will be discussed. The third chapter of the study provides details about data collection about augmented reality advertising, health consciousness, innovation resistance, interest in extreme sports, sampling technique, and population, and then, the data validity will be analyzed. In the fourth chapter, the results of the study will be presented. The study implications and the conclusion will be presented in the fifth chapter. Besides that, this chapter will also provide the study conclusion and future recommendations for scholars.

## Literature review and hypothesis development

The existence of television has introduced various methods of enriching the quality of image among people. There is a variety of broadcast imaging that is usually helpful for creating interest in people to watch more than their requirements or needs. People all around the world watch television due to their interest in sports, and in this, augmented reality advertising plays a vital role. In this context, (12) discussed the impacts and dominance of augmented reality advertising around the world, increasing motivation, and capturing people's attention. The augmented reality varies with the levels of visuals endorsed during the videos as sensory information. Businesses and companies often use augmented reality advertising to introduce their new products to the market. Many retailers and organizations use augmented reality advertising to promote their services and products, further enhancing the experience of people in the market.

Furthermore, Serfioti and Hunt (13) enumerated the effects of message designs and interactivity through augmented reality advertising for attaining positive customer responses. Augmented reality advertising is a vital tool for organizations and companies to capture the consumer market. There is a competitive advantage among organizations, and the integration of augmented reality advertising helps capture such interest. Most people are interested in extreme sports, and augmented reality advertising introduces strong pictures and images to such people. Many extreme sports exist in the world that attract the attention of people. Camping, motocross, mountain biking, and diving are the prominent highlighted extreme sports people want to try. Therefore, augmented reality advertising strongly impacts the interest in extreme sports. Moreover, Dai and Menhas (14) elaborated on augmented reality advertising videos as advertising campaigns that significantly impact the interest in extreme products. There is a variety of segmentation in sports that are positively upgraded among people by augmented reality advertising.

It is vital to introduce a feasible environment to create interest in extreme sports and create an excellent image for the people. It is possible when the augmented reality advertising strategy is positively introduced. Forgoing in view, Dai and Menhas (14) narrated the technology of augmented reality advertising through useful online video ads that involve consumer association for the interest in sports. It contains significant 2D and 3D objects that positively draw attention among people toward extreme sports. The augmented reality advertisement contains numerous corresponding factors influencing college students' interest in extreme sports. Most college students are fond of enjoying creative activities like extreme sports. These sports types are offered in projects and entertainment programs that create innovative thinking among college students. Therefore, the sports broadcast advertised through augmented reality applications enhances the transmission of information among college students. In furtherance, Hetland et al. (15) assessed the participation of students in physical activities and extreme sports due to the consistent increment in augmented reality advertising. Some setups for extreme sports are usually expensive, but the introduction of these setups developed the idea of augmented reality advertising. It not only generates attention among students about extreme sports but also develops compelling motivation for strategic implementation to customize its needs in the market. Most other video ads contain commercials for products and services, but the image of extreme sports gains attention. Extreme sports that are more interactive with the extreme sports are often exposed to augmented reality advertising.

Similarly, Mamansag (16) discussed the benefits and impacts of augmented reality advertising in the interest of extreme sports that affect students' interests. Many fans who watch from their homes attain the immersive experience of augmented reality advertising. Even though many college students are attracted to participating in extreme sports due to the better image of gameplay, the 3D graphics of extreme sports are more interactive and positively induced in college students. This inducement contains the potential practical possibilities of augmented reality advertising that shapes the interest in extreme sports. Thus, the hypothesis derived from the above discussion is as below:

**H1:** Augmented reality advertising significantly influences interest in extreme sports.

Many countries in the world are on the back due to a lack of technological innovation. This innovation contains augmented reality advertising that captures more advantages for college students. In this context, Datta (17) analyzed the adoption of technological innovation and its resistance, which inserted a moderating and dominant role in augmented reality advertising and sports apps. The countries lacking innovation and technological advancements in their television images and pictures are playing a leading role in the interest in extreme sports. Most developing countries with more potential in extreme sports are resisting innovation.

Furthermore, Immonen et al. (18) investigated the digital nudging and innovation resistance roles in decisions toward the interests of extreme sports and advertisement. The resistance to innovation has a dominating role in augmented reality advertising that helps create more interest in extreme sports. Augmented reality advertising is considered a vitally important organ of extreme sports and creates more interest in sports. Innovation resistance has a moderating impact on the relationship between augmented reality advertising and interest in extreme sports. Furthermore, Feng and Mueller (19) examined the resistance to change while implementing the innovation policy and the relationship between augmented reality advertising and extreme sports. People and college students show more interest in extreme sports and are admired for playing them. They consider their interest in extreme sports due to augmented reality advertising. College students' interest in extreme sports is disrupted in many ways due to the lack of training, broadcasting, marketing, betting, referring, and engagement. Governments all around the world are supporting extreme sports due to their creativity and attractiveness. Many students around the globe are inducing a more substantial interest in extreme sports due to the effective implementation of augmented advertising. In addition, Tsai et al. (20) numerous realities are related to augmented reality and virtual reality advertising in tourism for developing interest in extreme sports. On the contrary, countries' lack of resources and technology are considered the main hurdles in augmented reality advertising.

Therefore, the focus of innovation in extreme sports has been reduced in some countries due to their lower dominance. Moreover, Feng and Xie (21) explored the perspective of parents and children in extreme sports that are gradually increasing due to augmented reality advertising. The people and governments are also vital in enumerating the significance of innovation resistance. Augmented reality advertising strongly influences the interest in extreme sports, but the innovation resistance-moderating impact is also evident. In this context, Zhou et al. (22) discussed the injuries in extreme sports and adventures that have been widely enhanced with virtual and augmented advertising. The beliefs of people in emerging countries about technological advancement and innovation are majorly influencing people's interests. The moderating role of innovation resistance is crystal clear in the relationship between interest in extreme sports and augmented reality advertising.

Similarly, Raggiotto et al. (23) narrated the relationship between resistance to innovation, diversity, and competition paradoxes in extreme sports advertisements toward students. With the moderating effect of innovation resistance, uplifting, positive advertising can develop a wide range of opportunities. This advertisement promotes a positive image among the people and enables the students of developing countries to get acquainted with extreme sports. With the help of extreme sports, opportunity gates could be opened for international investors. Furthermore, Uhrich (24) analyzed the fitness of innovation resistance characteristics that play a vital role in augmented advertising and extreme sports machines. Many tourism countries are generating their most prominent revenue through extreme sports that have grown with augmented reality advertising. The awareness among developing countries facilitates augmented reality advertising due to the higher benefits and manages the innovation resistance. The role of innovation resistance provides a positive and negative picture of augmented reality advertising and extreme sports depending on the endorsement of the image. Thus, the hypothesis derived from the above discussion is as below:

**H2:** Innovation resistance significantly moderates the relationship between augmented reality advertising and interest in extreme sports.

Extreme sports are associated with leisure activities that also involve a large span of risk to the limbs and life. People and college students have always admired extreme sports, and their interest has increased due to the role of augmented reality advertising. In this context, Stryja and Satzger (25) analyzed consumers' choices due to their motivation toward health consciousness, social values, and environmental concerns over sports. Most youth students are convinced of extreme sports due to the considerable thrill-seeking element in their lives. The thrills in life contain immense risks of injury and death, but the narrative of augmented reality advertising has erased the safety measures.

Furthermore, Ranci and Arlotti (26) induced the integrated effects of habituation, self-efficacy, and health consciousness that have the dominant role in physical sports in college students have increased due to augmented reality advertising. People are more admired for taking the initiative in extreme sports possible due to augmented reality advertising. Augmented reality advertising has posed a positive image of videos through the 2D and 3D imaginary analysis that is most powerful for the thoughts of youngsters. Health consciousness is also essential between the corresponding elements of augmented reality advertising and interest in extreme sports.

Furthermore, Yung and Khoo-Lattimore (27) investigated college students' health consciousness and lifestyle practices, validating the scores of augmented reality advertising and extreme sports in adults. It has a moderating role in the developing interests of college students in extreme sports due to augmented reality advertising. Many sports in the world contain life-threatening risks, and injuries have often been caused in extreme sports. These sports involve skiing and jumping, which mainly cause injuries and are also considered illegal in many countries. College students are less health-conscious and less focused on their life risks; as a result, they mostly indulge in extreme sports. In addition, Mei-Dan (28) explored technology integration in the education sector by introducing mobile augmented reality advertising to assert science interest among students. Many college students face mental disorders and psychological impacts in their lives due to their increased interest in extreme sports.

The interest in extreme sports has increased due to the consistent rise in augmented reality advertising. In this context, Caine (29) enumerated the association between the extremity of sports injuries and training volume that has been specialized and increased with health consciousness and augmented reality advertising. This rise has been admired and convinced young college students to create an interest in extreme sports, but the element of health consciousness remained hidden. Furthermore, Dakka (30) were assessed among adventurers and college students due to augmented reality advertising. This hidden element of health consciousness inserts a moderating role between augmented reality advertising and interest in extreme sports.

Furthermore, Chen et al. (31) explored democratization and health consciousness, attributing their moderating role in extreme sports and augmented reality advertising. Due to the rising interest in extreme sports, augmented reality advertising for health consciousness has inserted significant measures. These measures include precautionary measures for saving the lives of people involved in extreme sports and paying more attention to these sports. In addition, Shin et al. (32) discussed the relationship between health consciousness, physical activity, and sports participation by college students motivated by augmented reality advertising. In some aspects, health consciousness is a vital organ of human health in extreme sports due to the use of mental and physical limits. When these limits are tried and stretched in extreme sports, it helps college students think and develop more approaches in their minds related to their professional life.

Moreover, Hong and Chung (33) analyzed the need for health consciousness and its moderating role in increasing and promoting augmented reality advertising interest in extreme sports. Most college students are approached at certain stages where they enhance their management skills over the elements of fear. This fear usually prevails among students when they try out extreme objectives and aims. Therefore, the increased balance and developed confidence in dealing with the worst situations also become positive for them. This positivity gains a powerful sense of humility among the students due to the working of various muscles of the human body parts. Thus, the hypothesis derived from the above discussion is as below:

**H3:** Health consciousness significantly moderates the relationship between augmented reality advertising and interest in extreme sports.

## Literature review gap

Extreme sports are the newest trend in sports. Players enjoy participating in exciting sports during physical activities, which is a growing trend among nations' youth. According to Zhou et al. (22), in addition to physical sports, players want to participate in extreme sports. However, the motivation factor could increase sports players' participation in extreme sports. Further research should be conducted to expand knowledge on extreme sports by investigating and developing relationships between motivation factors and extreme sports to discover the best relevant factors for extreme sports.

The research (8) has covered the extreme sports trend until 2017, and further interest and implications from Chinese participants must be analyzed to forecast the country's current scenario. According to Xu et al. (9), the development of Chinese sports is increasing yearly, but there is still a consistent lack in the development of extreme sports. Furthermore, growth in the Chinese sports industry is highest in the east and lowest in the west. There is a need to develop a strategy that can be implemented in both the east and west regions and provide equal and sustainable development to the sports industry, particularly China's extreme sports industry. The study also emphasized the associative growth of the Chinese health and sports industries because both of these factors help to keep the body active and because health through sports is popular. The motivation factor influencing extreme sports participants to demonstrate their skill is sustainable health (14).

According to a study Tsai et al. (20) augmented reality advertising is the most effective form of advertising, but it is not used to its full potential. Augmented reality advertising is a new way to bring innovation to purchasing and consumption behaviors, as well as to lifestyles and hobbies. As a result, more emphasis should be placed on practical implementation and developing a link between augmented reality advertising and innovation. According to Feng and Xie (21), augmented reality marketing is the future of innovative advertising. Consumers are more interested in augmented reality, and viewers may become potential new customers.

The significance of innovation in the utilize ability of extreme sports was emphasized by Uhrich (24). According to the research, the boost to the sports industry is the opportunity seized by the industry by innovating sports activities from traditional to thrilling sports such as extreme sports. These sports contribute to economic development, the sustainability of the sports industry, and the rise of extreme sports. However, resistance to change in some societies poses a barrier to the development and advancement of extreme sports (25). There is actual innovation resistance in technology adoption when it comes to resistance to innovation. If the barrier to technology adoption can be removed, extreme sports in China will increase.

As a result of the above literature gap, there is a particular need to develop a link between extreme sports activities through technologically enabled innovation in augmented reality advertisement. With augmented reality advertising, China can equally adopt college youth from the east and west. It may be possible to provide generalizability of study findings with equal participation.

## Research methods

The study examines the impact of augmented reality advertising on college students' interest in extreme sports. Also, it investigates the moderating role of innovation resistance and health consciousness among the linkage of augmented reality advertising and interest in extreme sports of college students in China. The measurement of the variables is taken from the previously published study, and the variable augmented reliability advertising has a four-item scale as a measurement (34). In addition, the variable innovation resistance has eight-item scales as measurement extracted from Kalkbrenner (35).

Moreover, the variable health consciousness has six-item scales as a measurement taken from Sugimoto et al. (36). Finally, the variable interest in extreme sports has seven-item scales as a measurement extracted from Zimmermann and Andrieu (37). These measurements are given in Table 1.

**Table 1:** Variables and measurements.

**Items**	**Statements**	**References**
**Augmented reality advertising**		
ARA1	“Augmented reality is good for the advertisement purpose”	(34)
ARA2	“Augmented reality is favorable for the advertisement purpose”	
ARA3	“Augmented reality is positive for the advertisement purpose”	
ARA4	“Augmented reality is pleasant for the advertisement purpose”	
**Innovation resistance**		
RTI1	“I will wait to adopt new technology until it proves beneficial”	(35)
RTI2	“I need to clarify some queries and justify the reasons for adopting new technology”	
RTI3	“I am waiting for the right time and required capability to adopt new technology”	
RTI4	“I fear wasting my time using new technology”	
RTI5	“I need to get a solution for some of my complaints and objections before I adopt new technology”	
RTI6	“I fear certain changes in the organization may impose on me”	
RTI7	“Innovation is not for me”	
RTI8	“It is unlikely that I will adopt innovation in the future”	
**Health consciousness**		
HC1	“For better health, I often have been doing proper exercises at home”	(36)
HC2	“For better health, I often do gymnastics at home (such as dance, yoga, aerobics, etc.)”	
HC3	“For better health, I often walk around or jog around the house”	
HC4	“For better health, I often do stretching exercises at home (such as leg press, leg lift, joint movement, etc.)”	
HC5	“For better health, I often do housework at home (such as cooking and cleaning)”	
HC6	“For better health, I often do other exercises at home (such as tai chi, bodybuilding, strength training, etc.)”	
**Interest in extreme sports**		
IES1	“I love taking risks, although I know extreme sports may hurt me”	(37)
IES2	“Risk and extreme sports are inseparable”	
IES3	“I think the risk is one of the features that attracted me to extreme sports”	
IES4	“I love the moves which are risky in extreme sports more”	
IES5	“Excitement is an inseparable part of extreme sports”	
IES6	“I think extreme sports add excitement to my life”	
IES7	“The excitement I feel while doing extreme sports is incredible”	

**Figure 2:**
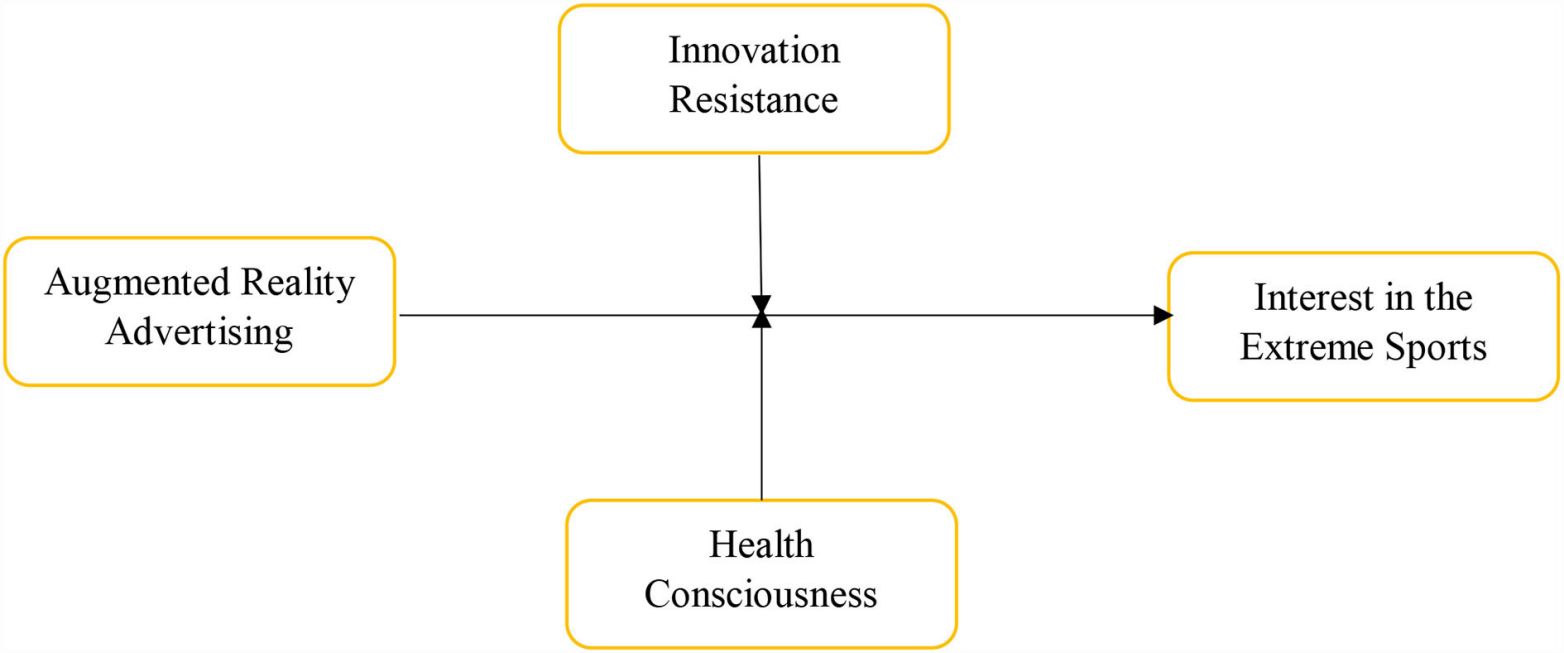
Theoretical model.

The study has selected the students who have participated actively in sports as the respondents. They were selected based on purposive sampling. The researchers have used primary data collection methods such as survey questionnaires to gather the primary data from selected students. These questionnaires were sent to the respondents using personal visits to the colleges. The researchers have chosen the top twenty colleges, and around 1,220 students who are attached with sports activities are selected. According to the Morgan sample size criteria, the sample size of the given population is 293. Thus, the researchers sent around 549 surveys to the students and received only 290 after 3 weeks. These surveys represent an approximately 52.82 % response rate. The study has only one predictor, such as augmented reality advertising (ARA). In addition, the study has taken two moderating variables named innovation resistance (IR) and health consciousness (HC). Finally, the study has taken only one predictive variable named interest in extreme sports (IES). These variables are mentioned in the framework in Figure 2.

## Conceptual framework

College students in China devote less time and attention to extreme sports. In this regard, the role of advertising is at the top of the list, and China's advertisement for extreme sports is not well-known or popular. In this regard, the framework developed in this study will aid in understanding the interest in extreme Chinese sports as well as the impact of advertising.

To generate public interest in extreme sports, it is critical to creating a viable environment and a positive image. If the augmented reality advertising strategy is successful, it is feasible. Most college students are drawn to artistic pursuits like extreme sports, and these activities are part of projects and entertainment content designed to encourage college students to think creatively. As a result, augmented reality apps that promote sports telecasts help college students improve data communication.

Because of a lack of technological innovation, many countries worldwide are falling behind. This innovation includes augmented reality advertising, which capitalizes on college students' advantages. Extreme sports are gaining popularity in countries with a lack of innovation. Most developing countries with higher potential in extreme sports are resistant to change. This resistance to change is evident in augmented reality advertising, which contributes to increased interest in extreme sports. Innovation resistance moderates the relationship between augmented reality advertising and interest in extreme sports.

Health consciousness is another factor influencing the relationship between augmented reality advertising and interest in extreme sports. Due to the growing popularity of extreme sports, augmented reality advertising for health awareness has advanced significantly. Precautionary measures to save participants' lives, as well as increased attention to these sports, are among these measures. It is an important aspect to be considered while participating in extreme sports due to the mental and physical use of the body.

The article has also applied AMOS to check the association among the variables and test the hypotheses. This tool works with large and small data sets and deals with complex models (38). In addition, it used two models: the measurement model to assess the validity and reliability and the structural model to check the association among variables. The measurement model assesses convergent reliability and discriminant validity. The convergent validity has been examined with the help of factor loadings, maximum shared variance (MSV), average squared shared variance (ASV), and average variance extracted (AVE), while the reliability has been examined using composite reliability (CR) analysis. In addition, the discriminant validity has been examined using the Fornell-Larcker criteria. The thresholds for factor loadings and AVE are that the values should be more than 0.50, while the thresholds for MSV and ASV are that the values should be lower than AVE (39).

SEM is performed using two tools: PLS and AMOS. When the sample size is small, <300, the SEM-PLS analysis is performed. Another use for SEM via PLS is when a researcher wants to investigate a new theory. The SEM-PLS is the best option for researchers when developing theories or developing new theories in their research. However, the reason for using SEM via AMOS, in this case, is that it handles large problematic sample sizes more efficiently than any other analyzing tool. SEM-AMOS is used by researchers to validate theoretically tested models. This study's framework is being tested to confirm the perceived and hypothesized relationships between the variables. SEM-AMOS is the best option for research testing when the goal of the research is to test theoretically proven variables in different situations and determine the applicability of the relationship.

Moreover, the standard criteria for CR are that the values should be more than 0.70, while the standard criteria for Fornell-Larcker are that the first value should be larger than the rest of the values in the column. In addition, the study has also checked the model's good fitness using root mean square error of approximation (RMSEA), comparative fit index (CFI), and Tucker–Lewis index (TLI). The standard criteria for RMSEA are that its values should be lower than 0.05 or between 0.05 to 0.10, while the thresholds for CFI and TLI are that the values should be more than 0.90 (40). Finally, the structural model assesses the linkage among variables using *t*-values and *p*-values. The standard criteria are that the *t*-values should be more significant than 1.64, and the *p*-values should be lower than 0.05. These tests are applied in the next section of the study.

### Research findings

**Figure 3:**
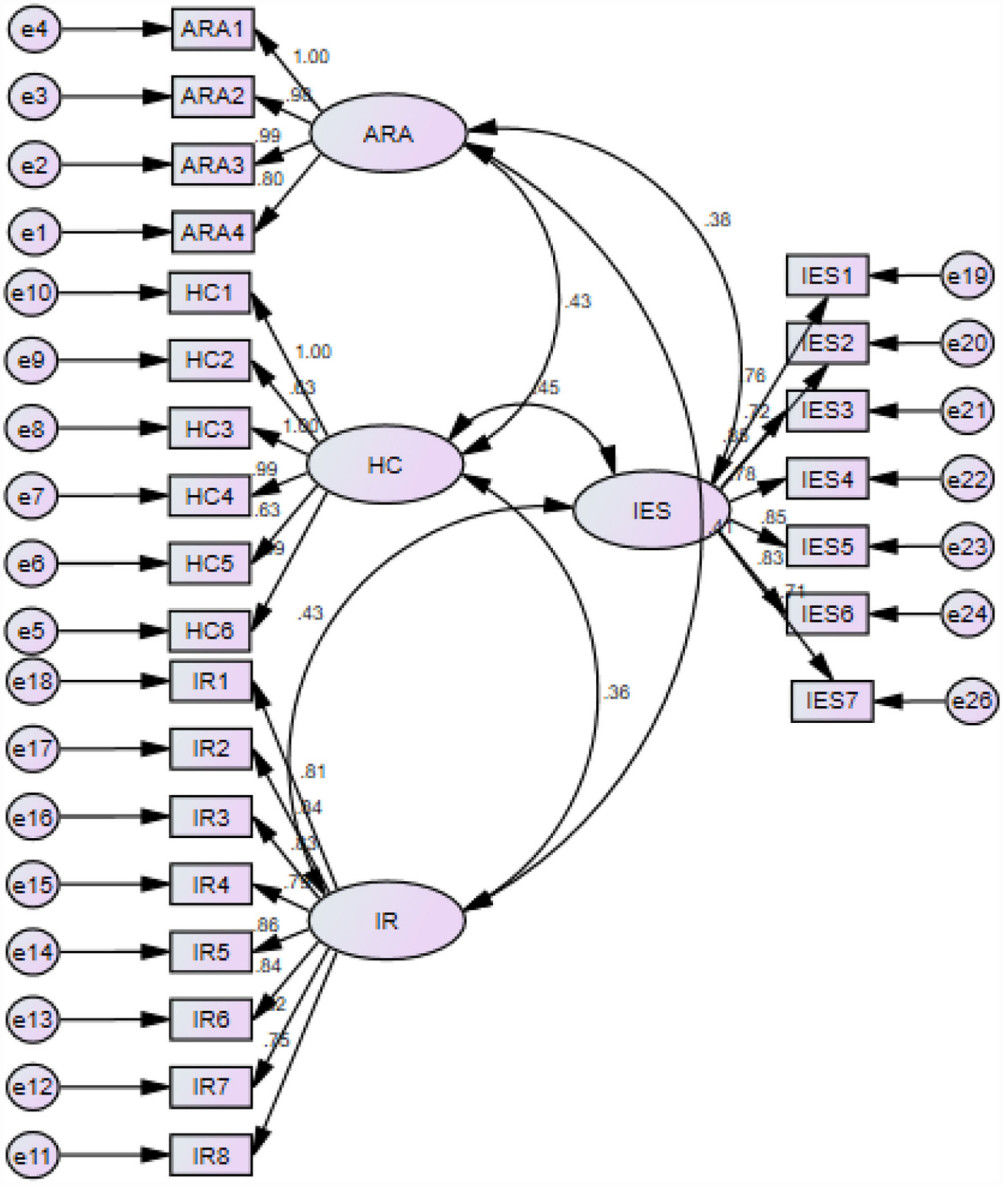
Measurement model assessment.

**Figure 4:**
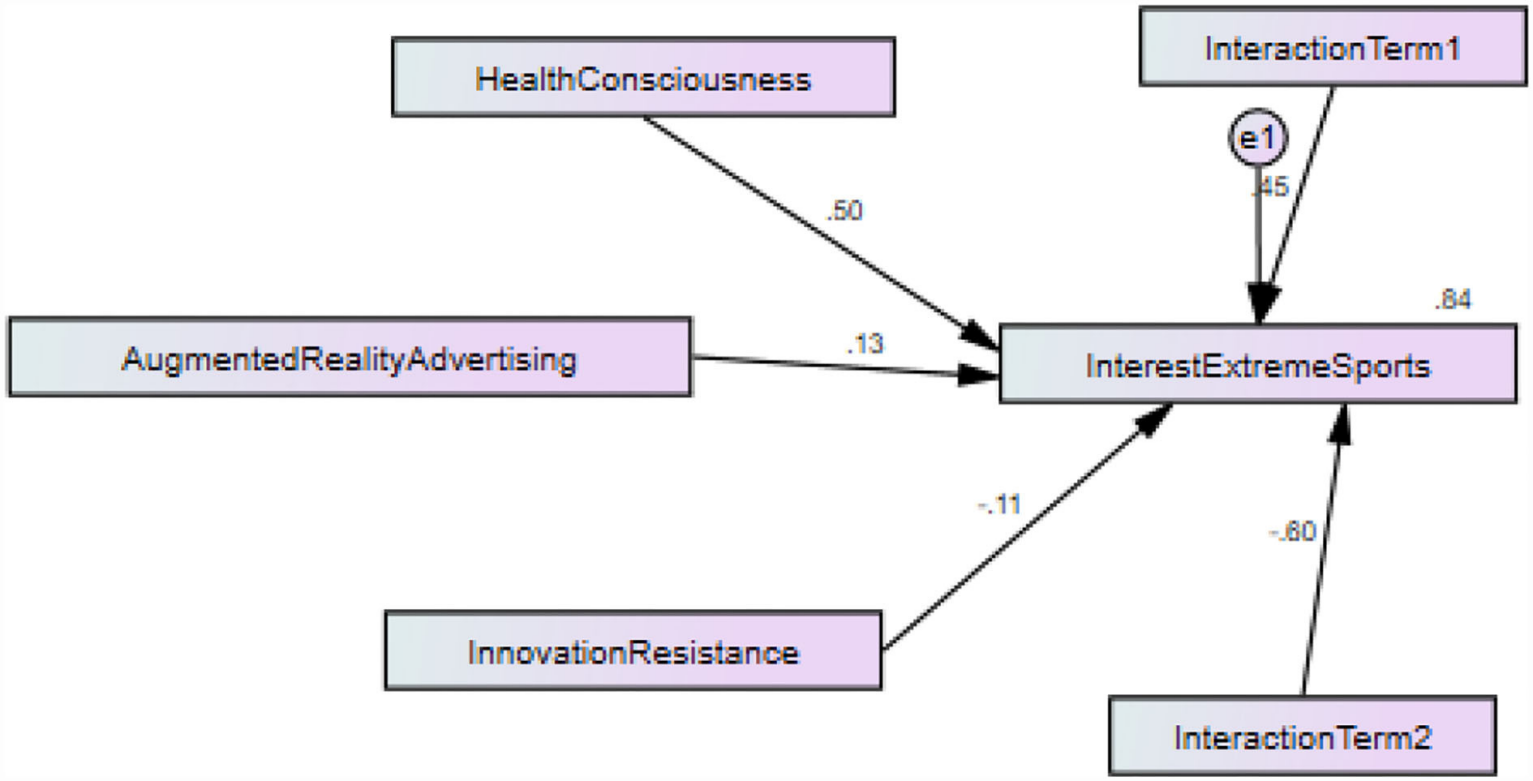
Structural model assessment.

The results show the convergent validity that exposed that item's correlation. The convergent validity has been examined with the help of factor loadings, MSV, ASV, and AVE. The thresholds for factor loadings and AVE are that the values should be more than 0.50, while the thresholds for MSV and ASV are that the values should be lower than AVE. The results indicated that the AVE and factor loading values are more significant than 50, and AVE values are more prominent than MSV and ASV values. Thus, the results exposed valid convergent validity. In addition, the study has also examined reliability using CR analysis. The standard criteria for CR are that the values should be more than 0.70, and the results also exposed that the CR values are more significant than 0.70. These results exposed good reliability, which is given in Table 2. Figure 3 shows measurement model assessment. Whereas Figure 4 shows structural model assessment.

**Table 2:** Convergent validity.

**Constructs**	**Items**	**Loadings**	**CR**	**AVE**	**MSV**	**ASV**
Augmented reality advertising	ARA4	0.800	0.942	0.670	0.182	0.159
	ARA3	0.992				
	ARA2	0.984				
	ARA1	1.000				
Health consciousness	HC6	0.993	0.972	0.898	0.189	0.167
	HC5	0.632				
	HC4	0.991				
	HC3	0.996				
	HC2	0.634				
	HC1	0.997				
Innovation resistance	IR8	0.746	0.957	0.793	0.202	0.173
	IR7	0.819				
	IR6	0.842				
	IR5	0.859				
	IR4	0.791				
	IR3	0.833				
	IR2	0.841				
	IR1	0.810				
Interest in extreme sports	IES1	0.760	0.919	0.619	0.202	0.177
	IES2	0.716				
	IES3	0.846				
	IES4	0.784				
	IES5	0.845				
	IES6	0.831				
	IES7	0.712				

In addition, the discriminant validity has been examined using the Fornell-Larcker criteria. The standard criteria for Fornell-Larcker are that the first value should be larger than the rest of the values in the column. The results also exposed that the first value is higher than the other values in the column and exposed valid discriminant validity. These outcomes are given in Table 3.

**Table 3:** Discriminant validity.

	**IR**	**ARA**	**HC**	**IES**
IR	0.818			
ARA	0.406	0.948		
HC	0.360	0.435	0.890	
IES	0.427	0.382	0.449	0.787

In addition, the study has also checked the model's good fitness using RMSEA, CFI, and TLI. The standard criteria for RMSEA are that its values should be lower than 0.05 or between 0.05 and 0.10, while the thresholds for CFI and TLI are that the values should be more than 0.90. The results indicated that the TLI and CFI values are higher than 0.90, and the RMSEA value is lower than 0.05. These outcomes are given in Table 4.

**Table 4:** Model good fitness.

**Selected indices**	**Result**	**Acceptable level of fit**
TLI	0.912	TLI > 0.90
CFI	0.919	CFI > 0.90
RMSEA	0.009	RMSEA <0.05 good; 0.05 to 0.10 acceptable

The structural model results revealed that augmented reality advertising has a positive link with interest in extreme sports because the beta has a positive sign and a significant linkage with interest in extreme sports. After all, *t*-values are more extensive than 1.64, and *p*-values are lower than 0.05 and accept H1. In addition, the results also revealed that if one % rise in augmented reality advertising, the interest in extreme sports also rises by 0.125 % and vice versa. The findings also indicated that innovation resistance and health consciousness significantly moderate among augmented reality advertising and interest in extreme sports of college students in China because *t*-values are more significant than 1.64 and *p*-values are lower than 0.05 and accept H2 and H3. These linkages are given in Table 5.

**Table 5:** Path analysis.

**Relationships**			**Std. Beta**	**Beta**	**SE**.	**CR**.	***P***
IES	< –	ARA	0.125	0.170	0.032	5.320	^***^
IES	< –	HC	0.499	0.759	0.036	21.173	^***^
IES	< –	IR	−0.114	−0.192	0.040	−4.829	^***^
IES	< –	Interaction Term 1	0.454	0.120	0.006	19.244	^***^
IES	< –	Interaction Term 2	−0.596	−0.153	0.006	−25.270	^***^

*** Means variable is significant at 1%.

## Discussions

According to the findings, augmented reality advertising has a positive relationship with developing interest in extreme sports. There have been numerous technological advancements in China. Digital technologies are constantly evolving, and augmented reality technologies have been developed (14). has discussed the impact of augmented reality advertisements as a powerful source of information for extreme sports. Various aspects of sports can be used in advertisements to create motivation and inspiration for athletes and sports players interested in extreme sports. The use of augmented reality advertisements aids in the promotion of the positive aspects of extreme sports. These technologies are critical in advertising because augmented reality allows for an interactive experience of world realities, which provides real-like satisfaction to humans' five senses and influences their thinking and emotions. The advertisement of extreme sports through augmented reality gives students a realistic feel and arouses excitement, interest, and the desire to participate in extreme sports. As a result, augmented reality advertising piques students' interest in extreme sports. These findings are supported by Yang et al. (41), which investigates the role of augmented reality adoption in promoting extreme sports. According to the study, companies that organize extreme sports such as bungee jumping, skydiving, scuba diving, rock climbing, base jumping, skiing, ice climbing, snowboarding, kayaking, skateboarding, and zip lining use augmented reality to advertise the games. Adolescents are inspired by augmented reality advertising and develop a desire to participate in extreme sports. These results are also in line with (42). This study sheds light on the role of augmented reality advertising in increasing people's interest in extreme sports. According to the study, the real-life auditory and visual experience of any event of games in extreme sports via augmented reality can better arouse people's interest in extreme sports, trigger excitement, and create an urge to play that game. These results also agree with (43), which emphasizes that augmented reality allows users to create images, videos, or live streaming in which many other things that do not exist in reality at the time can be added to the context. Using augmented reality, personnel in the advertising and marketing department can instill desired emotions and attitudes in the audience while also shaping their behavior. In extreme sports, the use of augmented reality can develop an interest in games in adolescents or students, diverting their attention away from the danger and toward the excitement. The results also match with (44), whose focus is on the role of augmented reality advertising and the growing popularity of extreme sports. According to this study, students or individuals become more interested in extreme sports in countries where augmented reality is used for advertising games.

The findings revealed that resistance to change is a significant moderator of augmented reality advertising in increasing interest in extreme sports. Although China is a rapidly emerging economy making technological advances, many educational, athletic, and other economic and social institutions fail to embrace innovation due to a lack of financial resources and weak policies. Furthermore, Uhrich (24) there is an element of innovative resistance that creates an irresistible spark in viewers and extreme sports enthusiasts. The validated relationship advertisement has a significant impact on their minds and creates a strong influence on them to participate. Resistance to innovation creates roadblocks in augmented reality advertising.

Furthermore, resistance to innovation prevents those around them from developing creativity and turning their attention toward something new, and as a result, they are unlikely to be interested in extreme sports. In this way, lowering resistance to change improves the role of augmented reality advertising in increasing interest in extreme sports. These results are supported by Purwanto et al. (45), emphasizing augmented reality advertising innovation and students' interest in extreme sports.

According to the study, the firms that want to advertise their products and services and the target audience for the advertisement place a high value on innovation adoption. It necessitates the use of cutting-edge technologies and apps. Effective augmented reality advertisements are not possible if there is resistance to innovation adoption on any side, whether by firms making advertisements or the target audience. Individuals cannot develop an interest in extreme sports in this situation of resistance to innovation. These results are also in line with (46), which demonstrates that if firms involved in the management of extreme sports and their participants do not adopt innovative digital technologies, they will be unable to benefit from augmented reality advertising to introduce the games and develop an interest in extreme sports. The message conveyed through augmented reality advertisements cannot reach individuals if societal resistance to innovation adoption. Individuals' extreme sports interests cannot be separated. These results also align with (47), which states that resistance to innovation coupled with the lack of improved digital technologies makes it difficult to use augmented reality for commercial purposes. The resistance to innovation becomes a barrier to developing awareness of games based on extreme sports, the associated precautions from dangers, and the excitement involved; so, interest development for extreme sports is complex. Hence, if there is a reduction in resistance to innovation, augmented reality advertising helps develop an interest in extreme sports.

The results showed that resistance to innovation is a significant moderator between augmented reality advertising in developing interest in extreme sports. These results are supported by Loia and Orciuoli (48), which demonstrates that many people who are more health conscious think deeply before deciding and acting. Such people prefer personal visual, auditory, or interactive experiences rather than relying solely on images, videos, and streaming, which are thought to conceal health-damaging elements. Furthermore, extreme sports include both danger and excitement. When people have a high level of health awareness, they are aware of the health risks and are less likely to be drawn to extreme sports. As a result, effective results from augmented reality advertising for developing interest in extreme sports can be obtained if people are informed about the precautions. These results are also in line with (49), which contends that if the target audience has a high level of health consciousness, they may be confused about the validity of augmented reality advertisement clips or pictures and may not be influenced by them. Furthermore, health consciousness prevents people from becoming interested in dangerous extreme sports. These results also match with (50), which suggests that people's consciousness influences the effectiveness of augmented reality advertising and students' interest in extreme sports and thus influences the relationship between augmented reality advertising and students' interest in extreme sports.

## Implications

### Theoretical implications

This study has theoretical and empirical implications. This study is notable in the literary world for its significant contribution to sports literature. This research looks into the role of augmented reality advertising in increasing interest in extreme sports. Previously published research examined the role of augmented reality advertising in the marketing of manufacturing products or services related to human feeds or facilities. This study adds to the literature by investigating the role of augmented reality advertising in sports, particularly extreme sports. Previous research has looked into the impact of innovation resistance and health consciousness on augmented reality advertising and people's interest in extreme sports. However, nowhere has the role of innovation resistance and health consciousness in the relationship between augmented reality advertising and the growing interest in extreme sports been studied. This study investigates the role of innovation resistance and health consciousness as moderators in the relationship between augmented reality advertising and growing interest in extreme sports. One of the study's outstanding contributions is investigating the relationship between these factors in the Chinese economy.

### Practical implications

This study is significant in emerging economies where sports, particularly extreme sports, are the primary source of income because it focuses on how to develop an interest in extreme sports among the college-age population. The promotion of extreme sports increases employment opportunities, income levels, and tourism growth opportunities and develops many characteristics such as confidence, bravery, courage, and creativity. As a result, this study is critical, as it provides guidelines for advertising and promoting extreme sports. This study can guide entities that are introducing or participating in extreme sports such as bungee jumping, skydiving, rafting, surfing, scuba diving, rock climbing, base jumping, skiing, paragliding, ice climbing, snowboarding, hang gliding, kayaking, skateboarding, caving, and zip line. They must develop a policy for using augmented reality to promote extreme sports to pique adolescents' interest.

Economists and the government must develop and implement policies to encourage economic innovation, promote augmented reality advertising, and develop students' interest in extreme sports. This research assists regulators in developing regulations to increase interest in extreme sports through augmented reality advertising and the adoption of new technologies. Furthermore, policies concerning augmented reality advertising and the development of interest in extreme sports must be developed, keeping in mind that the public may have a high level of health consciousness and that by promoting health precautions and safety, augmented reality advertising can be promoted, as can adolescents' interest in extreme sports.

## Conclusion

This study sought to investigate the effects of augmented reality advertising on the development of interest in extreme sports. The study also sought to investigate the role of innovation resistance and health consciousness in the relationship between augmented reality advertising and growing interest in extreme sports. A questionnaire-based empirical survey of the Chinese economy was conducted to collect data on augmented reality advertising, innovation resistance, health consciousness, growing interest in extreme sports, and their relationship. The findings revealed a link between augmented reality advertising and interest in extreme sports. The findings revealed that augmented reality allows for the presentation of extreme sports as they are played in real life, right in front of someone's eyes. The ability to have a real-life audio and visual experience of any extreme sports game via augmented reality may better awaken people's interest in extreme sports, induce enthusiasm, and inspire a desire to play that game. According to the findings, using augmented reality for advertising is difficult due to resistance to innovation, which leads to a lack of digital technology. As a result, developing an interest in extreme sports is difficult. As a result, augmented reality advertising can potentially increase interest in extreme sports if resistance to innovation decreases. According to the study, if the target audience has a high level of health awareness, they may be skeptical of the legitimacy of augmented reality commercial clips or images and thus may not be influenced by them. Furthermore, people's health consciousness keeps them from participating in potentially dangerous extreme sports.

### Limitations and future recommendations

Just like other studies, this article has many limitations. Future authors are recommended to remove these limitations with their research expertise. First, the study examines the role of augmented reality advertising in creating interest in extreme sports. The factors such as information and communication technologies, individuals' access to extreme sports, social status, and safety elements also have to do with the rise in students' interest in extreme sports. Using a single factor to determine the interest in extreme sports presents the study as incomplete or less comprehensive. It is up to future authors that they should not only focus on a single factor for the analysis of interest in extreme sports. Innovation resistance and health consciousness are the two mediators in this study, which have been analyzed as a link between augmented reality advertising and students' interest in extreme sports. It is recommended to future authors that they must also check the impacts of at least one mediator between augmented reality advertising and interest in extreme sports. The authors have analyzed the relationship between augmented reality advertising with interest in extreme sports and the relationship between innovation resistance and health consciousness with augmented reality advertising and interest in extreme sports in light of evidence from China. The data collected from a single economy for numerating interest in extreme sports cannot be a valid study for other countries on equality. That is why it is expected that future researchers must analyze these factors' roles in developing an interest in extreme sports.

## Data availability statement

The original contributions presented in the study are included in the article/supplementary material, further inquiries can be directed to the corresponding author/s.

## Ethics statement

The studies involving human participants were reviewed and approved by the Hubei Leisure Sports Development Research Center, China. The patients/participants provided their written informed consent to participate in this study.

## Author contributions

SZ conceived the idea. NH designed and wrote the paper. All authors read and agreed to the published version of the manuscript.

## Conflict of interest

The authors declare that the research was conducted in the absence of any commercial or financial relationships that could be construed as a potential conflict of interest.

## Publisher's note

All claims expressed in this article are solely those of the authors and do not necessarily represent those of their affiliated organizations, or those of the publisher, the editors and the reviewers. Any product that may be evaluated in this article, or claim that may be made by its manufacturer, is not guaranteed or endorsed by the publisher.
